# Urine-Based Molecular Diagnostic Tests for Leishmaniasis Infection in Human and Canine Populations: A Meta-Analysis

**DOI:** 10.3390/pathogens10030269

**Published:** 2021-02-27

**Authors:** Styliani A. Pappa, Panagiota I. Kontou, Pantelis G. Bagos, Georgia G. Braliou

**Affiliations:** Department of Computer Science and Biomedical Informatics, University of Thessaly, 2-4, Papasiopoulou Str., 35131 Lamia, Greece; spappa@uth.gr (S.A.P.); pkontou@uth.gr (P.I.K.); pbagos@compgen.org (P.G.B.)

**Keywords:** leishmaniasis, urine, molecular, diagnosis, meta-analysis, infection, PCR, sensitivity, canine, human

## Abstract

Leishmaniasis is a neglected tropical disease affecting humans and domesticated animals with high mortality in endemic countries. The pleiotropy of symptoms and the complicated gold-standard methods make the need for non-invasive, highly sensitive diagnostic tests imperative. Individual studies on molecular-based *Leishmania* diagnosis in urine show high discrepancy; thus, a data-evidenced comparison of various techniques is necessary. We performed a systematic review and meta-analysis using the bivariate method of diagnostic methods to pool sensitivities and specificities. We investigated the impact of DNA-extraction method, PCR type, amplified locus, host species, leishmaniasis form, and geographical region. The pooled sensitivity was 69.2%. Tests performed with the kit-based DNA extraction method and qPCR outweighed in sensitivity the phenol-chloroform-based and PCR methods, while their combination showed a sensitivity of 79.3%. Amplified locus, human or canine as host and cutaneous or visceral leishmaniasis revealed similar sensitivities. Tests in European and Middle Eastern countries performed better than tests in other regions (sensitivity 81.7% vs. 43.7%). A combination of kit-based DNA extraction and qPCR could be a safer choice for molecular diagnosis for *Leishmania* infection in urine samples in European–Middle Eastern countries. For the rest of the world, more studies are needed to better characterize the endemic parasite species.

## 1. Introduction

Leishmaniases are vector-borne diseases caused by at least 54 protozoan parasite species of the genus *Leishmania*, 21 of which are pathogenic to humans. They are transmitted to the mammalian hosts by infected female sandflies [1,2]. Leishmaniasis is considered a neglected tropical disease that comprises a high disease burden in developing (and a few developed) countries. Approximately 0.7 to 1 million new cases occur annually in about 100 endemic countries [3]. Leishmaniases can be considered zoonoses, anthropozoonoses or anthroponoses, with few species reported to exclusively infect humans. The *Leishmania donovani* complex is hosted by a number of vertebrates, and especially by humans and dogs [2,4,5]. Distinct species may present distinct clinical manifestation; however, the most prominent forms of leishmaniasis are cutaneous (CL) and visceral (VL). The disease is displayed with a broad range of severity and symptoms ranging from asymptomatic to life threatening VL cases, all related to the infecting parasite, vector biology, genetic and immune status of the host, as well as co-infections [2,6]. Other risk factors include poverty, malnutrition, population displacement, poor housing or even wars [5].

Combating leishmaniases entails certain challenges: (i) a great proportion of the infected hosts are asymptomatic [3]; (ii) vaccine development is still puzzling [7]; (iii) severe adverse effects of the few drugs against *Leishmania* parasites and drug resistance development of parasite strains [8]; (iv) necessity to control the domestic animal reservoir of the parasite in endemic residential areas; and (v) diagnostic assays, so far, are primarily developed to evaluate clinical disease and not parasitic carriage. The intervention strategies taken by the WHO to prevent and control leishmaniasis include effective disease surveillance, early diagnosis, and prompt, effective treatment [5].

Gold standard methods for diagnosis comprise the microscopic examination of lesion samples (for CL), or spleen aspiration, bone marrow or lymphoid node biopsy (for VL) smears or cultures of the samples. Although these methods show high specificity, sensitivity varies and cannot discriminate distinct species of the parasite [2,9,10]. Other conventional diagnostic methods include immunological tools for diagnostic tests both in serum and urine samples, such as an immunefluorescent antibody test (IFAT) [11], a direct agglutination test (DAT) [12], antigen-capture ELISA [13], latex agglutination test (KATEX) [14], antibody-capture ELISA, and dip-stick test [15]. However, despite their high diagnostic accuracy, serological methods present some limitations in their use because the identification of antibodies in cured persons cannot distinguish between immunity and an ongoing infection in an asymptomatic individual; thus, they cannot always help in decisions for treatment [9].

With the advent of many *Leishmania* species genomes, PCR-based methods can help diagnosis to discriminate between *Leishmania* subgenera, complexes, and species, and help in the epidemiological studies of zoonotic, anthroponotic and anthropozoonotic species, and in species-specific prognosis and treatment [2]. Several biological samples have been used, including blood, bone marrow, spleen, lymph nodes, skin lesions, conjunctival swabs, and urine [9,16,17]. Despite the large variety of sample options, most of them require special procedures either for sampling or for laboratory diagnosis and can entail risk and discomfort for both patients or diseased pets and healthcare providers. Thus, urine sampling combined with PCR-based diagnosis emerges as an ideal tool for a safe and sensitive method with high potential for leishmaniasis surveillance, prognosis, and diagnosis [18,19].

Many studies have been performed with urine samples using PCR-based methods and yielded a broad range of sensitivities. It seems that before such a method is widely used, many issues have yet to be solved, such as DNA extraction methods [9]. The present meta-analysis is an effort to quantitatively estimate the effect of several methodological aspects in PCR-based methods applied to urine samples of *Leishmania*-infected subjects in terms of diagnostic accuracy and validity.

## 2. Results

### 2.1. Studies’ Characteristics

**Figure 1:**
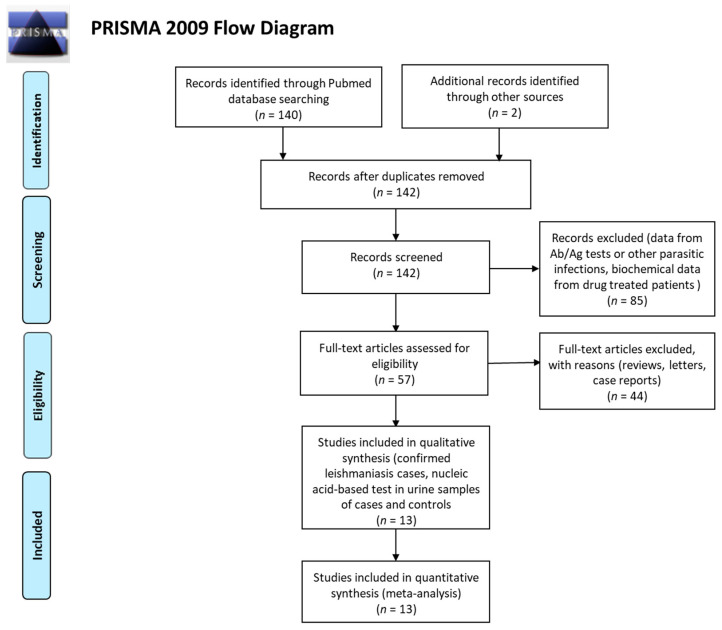
Preferred reporting items for systematic review and meta-analyses (PRISMA) flow diagram for the literature search.

With the primary search, we retrieved 140 articles from PubMed, Google Scholar, and Scopus. Scrutinizing lists of references added two more studies, while the removal of duplicates and compliance with eligibility criteria left us with thirteen studies (Figure 1). Nine studies were on human populations from various geographical regions, while four studies concerned cases of dogs from the Mediterranean Basin. In total, 367 cases were included, with 234 healthy controls. From these, 123 were diseased dogs and 13 were healthy canines. Ten studies provided data on cases and controls, and three studies only on cases (Table 1). Seven articles were on VL, six on CL, and one study reported data on both forms of *Leishmania*-infected patients. Eight studies were on populations from the Mediterranean Basin and Middle East, where the subspecies *L. infantum* is prevailing; four studies were on cases from South America; and one was on a Thai population. All studies reported that infection was ascertained by at least two different methods (microscopic diagnosis, parasite counts from tissue culture, PCR, Ag or Ab tests performed in skin lesions, blood, bone marrow aspirates or tissue biopsies) in accordance with standard protocols developed for the different forms of leishmaniasis [10,17]. In seven studies, the analysis of the *Leishmania* nucleic acid was performed with PCR (simple, nested, or followed by restriction fragment length polymorphism (RFLP) analysis), while six studies enrolled real-time quantitative PCR (qPCR). For DNA extraction from urine samples, phenol–chloroform based extraction method [20] was used in four studies, while seven kits from four different companies were reported in nine studies.

### 2.2. Analysis of Diagnostic Performance

**Figure 2:**
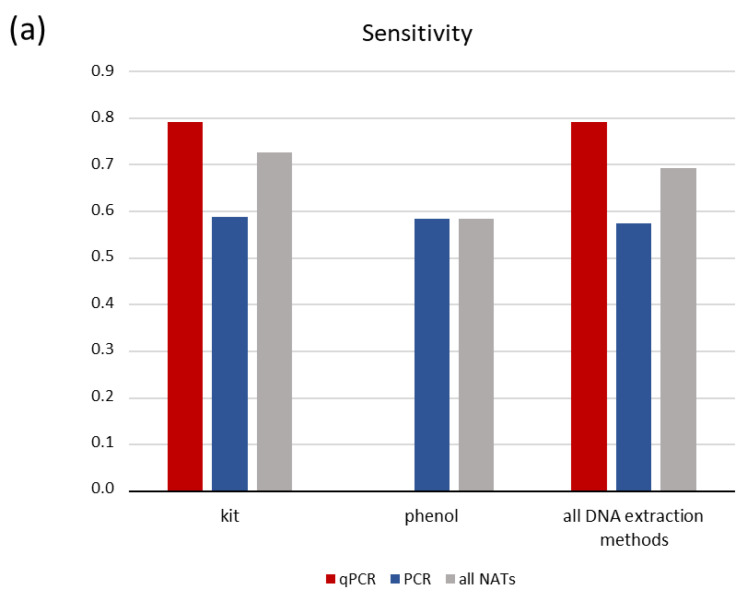

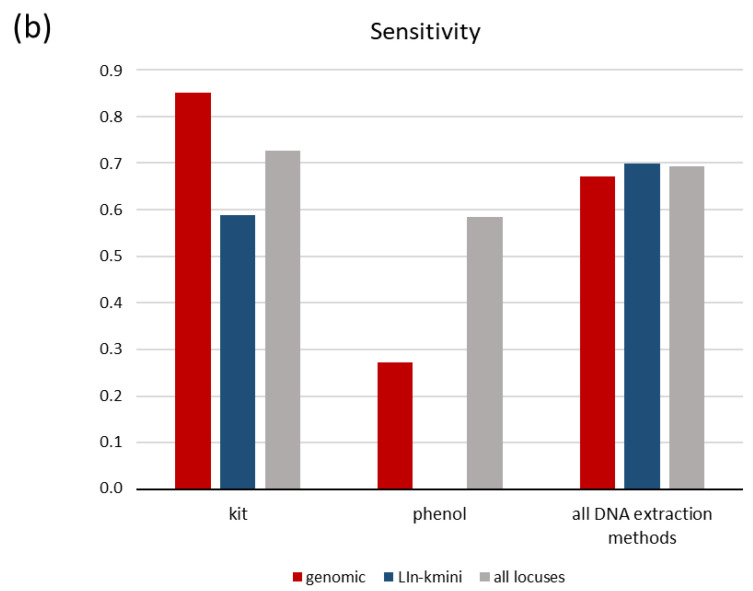
Pooled sensitivity of tests acquired from the meta-analysis according to DNA extraction method and (**a**) type of PCR or (**b**) amplified locus. NAT, nucleic acid test; *LIn*-kmini: *Leishmania infantum* or *Leishmania Viannia* k-minicircle DNA.

Our analysis was performed on all data collectively; however, more informative insight was gained when we grouped the tests according to different variables mentioned above. The overall sensitivity of all nucleic acid tests in urine samples from leishmaniasis patients is 0.692 (95% CI: 0.501, 0.883), while specificity is 1 (95% CI: 0.927, 1.000) (Table 2). Because a major concern of urine sampling is to obtain high quality and quantity of DNA for PCR amplification, we stratified our analysis according to molecular biology methodologies used for the analysis of the samples. Kit-based DNA extraction (nine studies) performed better as compared to phenol-chloroform-based DNA extraction (four studies) in terms of sensitivity, i.e., 0.728 (95% CI: 0.535, 0.917) as compared to 0.585 (95% CI: 0.234, 0.936) (Figure 2a and Table 2). Similarly, qPCR (six studies) seemed to perform better that simple, nested or RFLP combined PCR (seven studies), with a sensitivity of 0.793 (95% CI: 0.592, 0.993) as compared to 0.574 (95% CI: 0.326, 0.822). Because the simple PCR tests performed on phenol–chloroform-extracted DNA showed the same low sensitivity of 0.585 (95% CI: 0.234, 0.936) as the kit-PCR combination (0.588, 95% CI: 0.082–1.000), and because all qPCRs were performed on kit-based extracted DNA, we conclude that the combination of kit-based DNA extraction followed by qPCR (followed in six studies) performs better, with a diagnostic sensitivity of 0.793 (95% CI: 0.592, 0.993) (Figure 2a and Table 2). Another aspect concerning methodological issues is the locus amplified with PCR-based methods. We found that amplification of any genomic region (ITS1, ITS2 or another) presents sensitivity of 0.671 (95% CI: 0.379, 0.963), which is similar to the k-mini-circle DNA-based tests of 0.699 (95% CI: 0.473, 0.925), with six and seven studies respectively, as presented in Table 2. However, when combined with kit-based DNA extraction method, the sensitivities presented significant divergence: 0.851 (95% CI: 0.746–0.956) for the combination kit-genomic and 0.589 (95% CI: 0.311, 0.866) for the combination kit–*L. in*-kmini-DNA (*Leishmania infantum* k-minicircle DNA) (Figure 2b and Table 2). Similar sensitivity results were obtained when the kit-based method combined with qPCR and compared genomic and k-mini-circle loci outcomes, reinforcing the notion that the combined use of a kit for DNA extraction and qPCR performs best (Table 2). Specificity was 1 in all cases.

**Figure 3:**
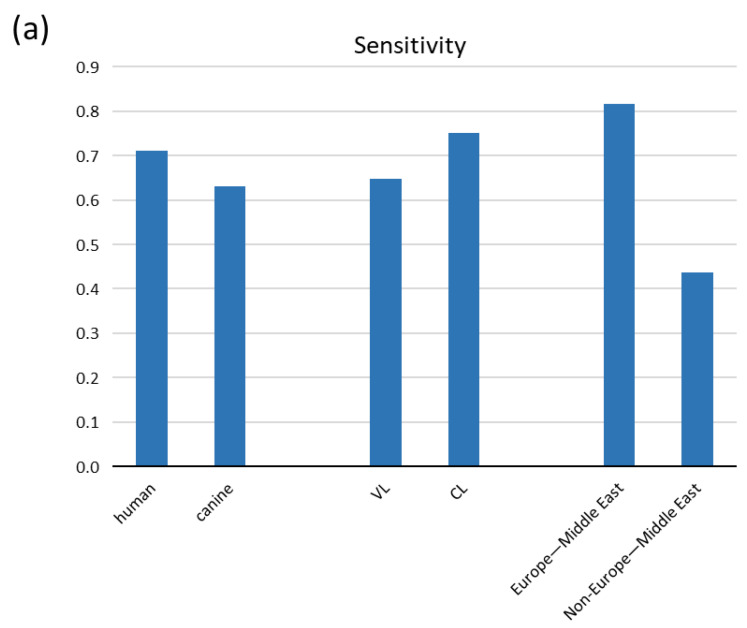

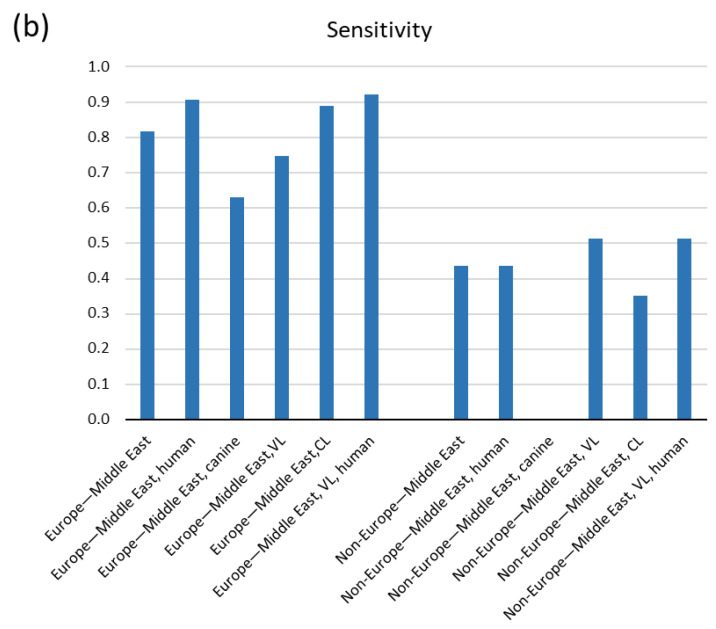
Pooled sensitivity of tests acquired from the meta-analysis according to (**a**) host, form of leishmaniasis and geographical region and (**b**) combinations of them.

Because canines can host human infecting *Leishmania* parasites, investigating canine cases was of high priority. Sensitivity estimates from the subgroup meta-analysis according to host species were also comparable, with human studies performing a bit better 0.712 (95% CI: 0.489, 0.934) compared to canines 0.631 (95% CI: 0.296, 0.967) (Figure 3a and Table 3). Although urine was first thought to be an additional sample for VL due to the high incidence of acute kidney injury (AKI) [32], diagnosis for CL has also effectively recruited urine sampling. We analyzed eight studies on VL and five on CL and found CL to perform a bit better with their respective sensitivities to be 0.649 (95% CI: 0.449, 0.849) and 0.751 (95% CI: 0.386, 1.116) (Figure 3a and Table 3).

In our meta-analysis we encompassed eight studies from the Mediterranean Basin and Middle East (Paleotropics), where the subgenus of *Leishmania* and species *L. infantum* and *L. donovani* prevail. We also included four studies from South America (Neotropics) where the *Viannia* subgenus predominates (*L. Viannia*) [4] and one study from Thailand where the subgenus *L. Mundinia* is endemic (species *L. siamensis* that reportedly is *L. martiniquiensis* [4]). Pooled analysis according to geographical region revealed a remarkable difference in sensitivity between Europe–Middle East and Non-Europe–Middle East locations, with the studies from Europe–Middle East showing a higher sensitivity of 0.817(95% CI: 0.639, 0.995) compared to the latter with a sensitivity of 0.437 (95% CI: 0.220, 0.653) (with eight and five studies, respectively) (Figure 3a and Table 3). The vast dependence of sensitivity on the geographical region of the samples is further depicted by subgroup analyses within the same regions and stratifying for VL/CL, human/canine or VL/CL and human/canine simultaneously (Figure 3b and Table 3). However, the last estimates arise from very few studies (two or three), and thus they denote high uncertainty. Remarkably, combination of the best qPCR, kit, genomic and Europe–Middle East resulted in the highest sensitivity of 0.888 (95% CI: 0.785, 0.992); however, this results from only two studies and denotes high ambiguity.

## 3. Discussion

*Leishmania* parasite counts from bone marrow, lymph node, skin lesions or even blood samples (smears or cultures) have long been the gold standard for either VL or CL diagnosis to dictate the onset of treatment. Specialized lab equipment and expert staff are mandatory, the sensitivity is rather low, and the procedure poses discomfort and risk to both subjects and health professionals [33]. An approach to surpass the above issues is the use of serological tests which show improved sensitivity and specificity and are less risky (for subjects and staff), however co-infections or past infections can mislead health professional to treatment strategy decisions [2,34]. In the 21st century, urine samples have gradually been used for the detection of cell-free circulating DNA (cfDNA) spanning 150 to 250 bp coming from apoptotic, cancerous, or infected cells [9,35]. *Leishmania* nucleic acid can cross the glomerular filtration barrier and can be found in urine. In addition, in VL patients, who very often suffer from acute kidney disease (AKI) which causes deposition of immune complexes to—and inflammation of—kidneys, *Leishmania* parasites may be released to the urinary track [9,32]. Urine has many advantages as a sample because it can be collected easily and several times with no discomfort or any risk for subjects or health professionals. Considering the high sensitivity usually offered by PCR-based techniques and the fact that it can be coupled to molecular typing of the parasite, it becomes clear that urine-based molecular biology diagnosis is a very promising approach to detect, quantify, identify the infecting *Leishmania* species and even predict clinical manifestation (if at the beginning of infection).

To this direction, studies have been performed to demonstrate and improve the usefulness of PCR-based diagnosis of *Leishmania* infection from urine samples [2,9,16]. The present meta-analysis is an effort to quantitatively summarize the evidence of all thirteen published studies comprising data from 367 infected and 234 non-infected subjects (humans and dogs). We included studies on both VL and CL subjects and we investigated many methodological aspects. The meta-analysis revealed that all PCR-based methodologies yielded high specificity (100%); in all studies, no false positives were found. The overall sensitivity of the molecular tests in urine samples is 69.2%. This meta-analysis revealed many methodological issues that could explain the low performance of this technique in some cases. DNA extraction methods have long been puzzling in such studies [9]; we demonstrated that kit-based DNA extraction from urine combined with qPCR significantly raised the sensitivity to 79.3%. It is not yet completely clear how—however well known—that substances and biological components of the urine can interfere with the PCR amplification reaction (especially in the urine of HIV infected patients) [23,36,37]. Precaution during storage and transportation, addition of preservatives, and high-speed centrifugation to eliminate intact cells that could affect the quality of isolated cfDNA have also been investigated in a good practice perspective [38]. Unfortunately, such comprehensive data were not available from each of the included studies and thus, further subgroup analysis could not be performed. Nine different set of primers have been used in the thirteen studies of the present meta-analysis covering various regions of either genome (six studies) or the k (kinetoplastid) minicircle DNA (seven studies) of the parasite. Genomic versus kDNA amplification approaches showed comparable sensitivity (67.1% vs. 69.9%).

Given that leishmaniasis is an anthropozoonosis, the prevalence, the preservation and dispersion of the disease is highly related to domestic animal reservoirs of the parasite [4]. Only four studies offered data on canines (compared to nine for humans); sensitivity measures were comparable to those from human studies, with 63.1% for dogs and 71.2% for humans, suggesting that the methodology could be equally well adopted for animals. Thus, the present results encourage more animal studies to be performed, so that valuable information is added. Similarly, subgroup analysis according to the form of leishmaniasis resulted in comparable sensitivities of 64.9% for VL and 75.1% for CL, again suggesting that urine sample is equally appropriate for cutaneous infection diagnosis, not really depending on kidney injuries (often occurring in VL) but rather on the crossing of the glomerular barrier of the small DNA molecules of the parasite [9,32]. Our findings support the increasing awareness that subdivision by form of leishmaniasis is rather ineffective for understanding and combating leishmaniasis, while the most intention should be given to the various species *per se* and their clinical manifestations in the vertebrate hosts [2].

The most studied subgenus of *Euleishmania* is that of *Leishmania* that includes four complexes, *donovani*, *major*, *tropica* and *mexicana*. Eight studies were performed in countries of the Mediterranean Basin and Middle East, where *L. infantum* is endemic and prevailing, and the PCR primers used therein were for *L. infantum*. Four studies were performed with patients from Brazil, and Peru, i.e., in countries of South America, where *L. Viannia* predominates and *L. infantum* is also present. One study was from Thailand, and *L. siamensis* (*L. Mundinia*) was amplified. Interestingly, subgroup analysis according to geographical region revealed a substantial difference in sensitivity measures, being 81.7% for Europe–Middle East and 43.7% for the Non-Europe–Middle East countries. An explanation could be the fact that *L. infantum* is best studied and present in the former countries, while in the other countries the existence of multiple or even hybrid species may render parasite identification difficult. Moreover, the lack of in-depth analysis of *L. Mundinia* and insufficient sampling of *L. Viannia* results in less available experimental and genomic information for these subgenera [2]. More studies investigating various species with multiple sets of primers in the neotropical regions are of absolute need.

Our meta-analysis has also some limitations. Different ways of urine preservation (temperature, preservatives, and transport), as well as the non-defined time between reference standard performance and urine PCR in some studies, might have affected the stability of the assay and have introduced potential bias, as was shown in the quality assessment of the research performed with the QUADAS tool. In addition, the fact that in most studies, urine PCR/qPCR was performed after the knowledge of the results of other infection ascertainment methods, might also have introduced bias in our analysis as assessed with the QUADAS tool.

Molecular diagnostics of *Leishmania* infections using urine samples is a very promising tool, not only to help diagnosis and treatment prescription, but for epidemiological surveillance reasons as well. The results of the present meta-analysis suggest that molecular biology methodological aspects must be considered and propose the use of kit-based DNA extraction methods and qPCR. In addition, amplification of *L. infantum* kDNA and other genomic regions seem to perform with high sensitivity in European and Middle East countries. However, more studies, with attempts to amplify multiple regions of various endemic species in other geographical regions, are needed in order to raise the diagnostic performance of the method in these areas.

## 4. Materials and Methods

### 4.1. Literature Search Strategy

To conduct the present meta-analysis, preferred reporting items for systematic reviews and meta-analyses (PRISMA) guidelines [39], as well as advice for best practices, were followed [40]. The literature search was conducted in PubMed, Google Scholar and Scopus with the following search terms: leishmania and urine and (DNA OR polymerase OR PCR OR diagnostic OR diagnosis OR test) in October 2020. To include all published and unpublished studies (conference papers, dissertations), lists of references were examined to avoid grey literature publication bias [41,42].

### 4.2. Study Selection Criteria

We imposed no restrictions during the selection procedure (study design, language or other quality measures) [43]. Non-English manuscripts were also considered for review. Studies were excluded when they provided data on methods assessing antibodies, antigens, or other biochemical parameters in urine, serum, or other samples, or presented data on cases from other parasite infections. For a study to be eligible for the present meta-analysis, it had to meet the following criteria: (i) leishmaniasis cases had to be confirmed by at least two ways (parasitology, PCR, culture or antibody detection in tissues such as bone marrow, blood, skin lesions or aspirates); (ii) patients and controls could be either human or domestic animals; and (iii) studies had to report positive or negative results from urine samples performed with a nucleic acid-based test (NAT) in cases and controls. We also included as eligible studies those that only reported data of patients.

### 4.3. Data Extraction

Two researchers (S.P. and G.B.) extracted the data from each manuscript according to the eligible criteria. When problems of disagreement arose, they were resolved after discussion with a third reviewer (P.B.). Recorded data include the first author’s last name, year, species, geographic region of the participants, gold standard diagnostic method, type of leishmaniasis, method of DNA extraction from urine NAT used, genetic locus amplified, and comorbidity. To obtain the estimates for sensitivity and specificity, we created the 2 × 2 contingency table that included the numbers of true positive (TP), false positive (FP), true negative (TN), and false negative (FN) from each study. For studies reporting only leishmaniasis patients, only TP and FN were recorded.

### 4.4. Data Analysis

The quality of the included studies was assessed by the Quality Assessment of Diagnostic Accuracy Studies 2 (QUADAS-2 tool) according to the four domains: patient selection, index test, reference standard, and flow and timing (Tables S1 and S2, Figure S1). QUADAS is a tool to assess quality, developed for systematic reviews of studies on diagnostic accuracy [44]. To evaluate each domain, the following classifications according to judgment were used—low risk, high risk, and unclear risk.

The bivariate diagnostic meta-analysis was used [45], which is considered equivalent to the so-called hsROC method [46,47,48] to assess sensitivity and specificity measures. The analyses were performed with the “mvmeta” command with the method of moments for multivariate meta-analysis and meta-regression [49], using STATA 10 (Stata Corporation, College Station, TX, USA). *p* < 0.05 was set as the threshold for statistical significance and meta-analysis was performed for contrasts where two or more studies were available.

## 5. Conclusions

Diagnosis of leishmaniasis is performed in multiple ways depending on the sample type, the availability of lab infrastructure, and the objectives of the diagnosis which include treatment strategies and epidemiological surveillance. Urine comprises an easily taken, low risk sample. It is known that PCR-based parasite diagnosis offers high sensitivity and specificity. However, concerning leishmaniasis diagnosis, the published studies show high discrepancy concerning sensitivity which spans from 25% to 100%. This discrepancy makes the PCR-based methodology not directly applicable to leishmaniasis diagnosis. To the best of our knowledge, the present meta-analysis is the first attempt to systematically and quantitatively compare the effects of many methodological steps and other disease variables on the diagnostic performance of PCR-based diagnosis of leishmaniasis using urine samples. Our analysis demonstrates that kit-based DNA extraction (as compared to phenol–chloroform) from urine, combined with qPCR (as compared to simple PCR) performs remarkably better in terms of sensitivity of the diagnostic method. Moreover, the method performs better for cases from European and Middle Eastern regions as compared to other regions. A putative cause could be the fact that leishmaniasis has been extensively studied (genomic-wise) in Europe–Middle East countries, where *L. infantum* predominates. On the contrary, less genomic data are available from Leishmania species from other regions, highlighting the need for extensive investigation (covering more molecular DNA targets) for better biomarkers. Our research emphasizes the importance of not only detection but identification of the species in Leishmania infections that will help combat leishmaniasis worldwide in hospitals, health centers, and research institutes.

## Figures and Tables

**Table 1:** Characteristics of the Studies Included in the Meta-Analysis.

Author	Year	Country	Species	Comorbidity	Method of PCR	Amplified Locus	Form of Leishmaniasis	Leishmaniasis Ascertainment	DNA Extraction Method	Cases/Controls	Sensitivity	Specificity	TP/FN/TN/FP
Mirzaei [21]	2018	Iran	Human	NA	qPCR	ITS2-region of ribosomal DNA	CL	1. Blood-PCR 2. Serum-Westrn blot	Kit (QIAamp DNA Mini Kit QIAGEN)	23/3	0.869	1	20/3/3/0
Mirzaei [21]	2018	Iran	Human	NA	qPCR	ITS2-region of ribosomal DNA	VL	1. Blood-PCR 2. Serum-Westrn blot	Kit (QIAamp DNA Mini Kit QIAGEN)	14/3	0.928	1	13/1/3/0
Da Costa Lima Junior [22]	2018	Brazil	Human	NA	PCR/ RFLP	ITS1-region of ribosomal DNA	VL	1. ΒM aspirate, PB-PCR 2. Culture from LN, BM, spleen aspirates -Parasite counts 3.BM–Ab test ELISA, rk39-ICT	Phenol-chloroform	30/50	0.366	1	11/9/50/0
Pessoa-E-Silva [18]	2016	Brazil	Human	AIDS	qPCR	*L. infantum* kDNA minicircle	VL	1.Blood–qPCR 2. Blood, oral fluid-Ab test rK39–ICT	Kit (QIAamp DNA Mini Kit -QIAGEN RW Germany)	8/50	0.500	1	4/4/10/0
Almerice Lopes da Silva [23]	2014	Brazil	Human	NA	PCR	*L. infantum* kDNA minicircle	VL	1. BM, PB-PCR	Phenol–chloroform	11/NA	0.727	NA	8/3/-/-
Hernández [24]	2014	Spain	Canine	NA	qPCR	*L. infantum* kDNA minicircle	CL	1. BM, PB-Nested PCR, qPCR 2. Cultures from skin lesions, spleen, liver, BM, LN aspirates—Parasite counts 4. Serum–Ab test IFAT, ELISA	Kit (e QuiAamp DNA Micro kit QIAGEN)	8/NA	0.750	NA	6/2/-/-
Phumee [25]	2013	Thailand	Human	HIV/DMII	PCR	ITS1- gene of *L. siamensis*	CL	1. BM, blood, buffy coat tissue, saliva-PCR 2. Culture from blood, skin biopsy, BM-Parasite counts	Kit (Invisorb Spin Tissue Mini Kit)	5/4	0.600	1	3/2/4/0
Veland [26]	2011	Per-u	Human	NA	PCR/RFLP	*L. Viannia* kDNA minicircle	CL	1. Skin scraping aspirate-PCR 2. Culture from skin lesions-Parasite counts	Phenol–chloroform	86/22	0.210	1	18/68/22/0
Fisa [27]	2008	Spain	Human	AIDS in controls	Nested PCR	*L. infantum* genomic DNA	VL	1. Blood-PCR 2. Blood-ADU by KAtex 3. Culture from BM aspirates-Parasite counts	Kit (High Pure PCR Template Roche Molecular Biochemicals, Mannheim, Germany)	28/59	0.882	1	15/2/59/0
Motazedian [28]	2008	Iran	Human	NA	PCR	*L. infantum* kDNA minicircle	VL	1. BM, LN, spleen, PB, serum aspirates-PCR 2. Culture from LN, BM, spleen aspirate—Parasite counts	Phenol–chloroform	30/30	0.660	1	29/1/30/0
Manna [29]	2008	Italy	Canine	NA	qPCR	*L. infantum*, kDNA minicircle	CL	1. Serum–Antibodies test IFAT 2. LN aspirate–PCR	Kit (QIAamp blood QIAGEN Santa Ciarita, CA)	41/3	1.00	1	41/0/3/0
Solano-Gallego [30]	2007	Spain	Canine	NA	qPCR	*L. infantum* kDNA minicircle	VL	1. PB, BM, LN-PCR 2. Spleen aspirate, LN-Westrn blot, ELISA	Kit (High Pure PCR Templat Roche Applied Science)	43/10	0.465	1	20/23/10/0
Franceschi [31]	2007	Italy	Canine	NA	PCR	*L. infantum* kDNA minicircle	VL	1. LN-IFAT 2. LN-Parasite counts 3. Clinical signs	Kit (Accuprep Genomic DNA Extraction Kit Bioneer Korea)	40/NA	0.250	NA	10/30/-/-

PB: peripheral blood; BM: bone marrow; ICT: immunochromatographic test; IFAT: indirect fluorescent antibody test; ELISA: enzyme-linked immunosorbent assay; LN: lymph node; NA: not assessed; CL: cutaneous leishmaniasis; VL: visceral leishmaniasis; PCR: polymerase chain reaction; qPCR: quantitative PCR; RFLP: restriction fragment length polymorphism.

**Table 2:** Results of the Multivariate Meta-Analysis. Characteristics Concerning Methodological Issues are Presented with their Relative (and Pooled) Sensitivities and Specificities Estimates along with the Respective 95% Confidence Intervals.

DNA Extraction Method	Nucleic Acid Test	Locus Amplified	Studies/Patients/Controls	Sensitivity (95% CI)	Specificity (95% CI)
kit/phenol	qPCR/PCR	genomic/*L.in-kmini*	13/367/234	0.692 (0.501, 0.883)	1 (0.927, 1.000)
kit/phenol	PCR	genomic/*L.in-kmini*	7/230/165	0.574 (0.326, 0.822)	1 (0.956, 1.000)
kit/phenol	qPCR	genomic/*L.in-kmini*	6/137/69	0.793 (0.592, 0.993)	1 (0.816, 1.000)
kit	qPCR/PCR	genomic/*L.in-kmini*	9/210/132	0.728 (0.535, 0.917)	1 (0.880, 1.000)
phenol	qPCR/PCR	genomic/*L.in-kmini*	4/157/102	0.585 (0.234, 0.936)	1 (0.961, 1.000)
kit	PCR	genomic/*L.in-kmini*	3/73/63	0.588 (0.0816, 1.000)	1 (0.843, 1.000)
phenol	PCR	genomic/*L.in-kmini*	4/157/102	0.585 (0.234, 0.936)	1 (0.961, 1.000)
kit	qPCR	genomic/*L.in-kmini*	6/137/69	0.793 (0.592, 0.993)	1 (0.816, 1.000)
kit/phenol	qPCR/PCR	genomic	6/186/141	0.671 (0.379, 0.963)	1 (0.920, 1.000)
kit/phenol	qPCR/PCR	*L.in-kmini*	7/181/93	0.699 (0.473, 0.925)	1 (0.896, 1.000)
kit	qPCR/PCR	genomic	4/70/69	0.851 (0.746, 0.956)	1 (0.861, 1.000)
kit	qPCR/PCR	*L.in-kmini*	5/140/63	0.589 (0.311, 0.866)	1 (0.847, 1.000)
kit	qPCR/PCR	genomic/*L.in-kmini*	9/210/132	0.726 (0.535, 0.917)	1 (0.880, 1.000)
phenol	PCR	genomic/*L.in-kmini*	4/157/102	0.585 (0.234, 0.936)	1 (0.961, 1.000)
phenol	PCR	genomic	2/116/72	0.273 (0.111, 0.435)	1 (0.957, 1.000)
kit	qPCR	genomic	2/37/6	0.888 (0.785, 0.992)	1 (0.646, 1.000)
kit	qPCR	*L.in-kmini*	4/100/63	0.710 (0.417, 1.000)	1 (0.832, 1.000)

kit: kit-based DNA extraction method; phenol-chloroform-based DNA extraction method; qPCR: quantitative real time PCR; genomic: a genomic region was amplified (ITS1 or ITS2 or unspecified); *L.in-kmini*: *Leishmania infantum* or *Leishmania Viannia* k-minicircle DNA was amplified.

**Table 3:** Results of the Multivariate Meta-Analysis. Characteristics Concerning Methodological Issues are Presented with their Relative (and Pooled) Sensitivity and Specificity Estimates, Along with the Respective 95% Confidence Intervals.

Form of Leishmaniasis	Species	Geographical Region	Studies/Patients/Controls	Sensitivity (95% CI)	Specificity (95% CI)
VL	human/canine	all regions	8/204/202	0.649 (0.449, 0.849)	1 (0.945, 1.000)
CL	human/canine	all regions	5/163/32	0.751 (0.386, 1.000)	1 (0.856, 1.000
VL/CL	human	all regions	9/235/221	0.712 (0.489, 0.934)	1 (0.933, 0.999)
VL/CL	canine	all regions	4/132/13	0.631 (0.296, 0.967)	1 (0.814, 1.000)
VL/CL	human/canine	Europe–Middle East	8/227/108	0.817 (0.639, 0.995)	1 (0.904, 1.000)
VL/CL	human/canine	Non-Europe–Middle East	5/140/126	0.437 (0.220, 0.653)	1 (0.923, 1.000)
VL	human	all regions	6/121/192	0.775 (0.557, 0.994)	1 (0.948, 1.000)
CL	human	all regions	3/114/29	0.573 (0.007, 1.000)	1 (0.823, 1.000)
VL	human/canine	Europe–Middle East	5/155/102	0.748 (0.479, 1.000)	1 (0.926, 1.000)
VL	human/canine	Non-Europe–Middle East	3/49/100	0.514 (0.260, 0.768)	1 (0.931, 1.000)
CL	human/canine	Europe–Middle East	3/72/6	0.889 (0.748, 1.000)	1 (0.646, 1.000)
CL	human/canine	Non-Europe–Middle East	2/91/26	0.352 (0.068, 0.772)	1 (0.856, 1.000)
VL/CL	human	Europe–Middle East	4/95/95	0.906 (0.838, 0.975)	1 (0.911, 1.000)
VL/CL	human	Non-Europe–Middle East	5/146/126	0.437 (0.220, 0.653)	1 (0.923, 1.000)
VL/CL	canine	Europe–Middle East	4/132/13	0.631 (0.296, 0.967)	1 (0.814, 1.000)
VL	human	Europe–Middle East	3/72/92	0.923 (0.854, 0.997)	1 (0.935, 1.000)
VL	human	Non-Europe–Middle East	3/49/100	0.5138 (0.260, 0.768)	1 (0.931, 1.000)
CL	human	Non-Europe–Middle East	2/91/26	0.352 (0.068, 0.772)	1 (0.856, 1.000)

## Data Availability

The data used to support the findings of this study are included within the article.

## Supplementary Materials

The following are available online at https://www.mdpi.com/2076-0817/10/3/269/s1, Figure S1: The Quadas Tool 2 graph; Table S1: The QUADAS tool Item; Table S2: The Quadas Tool 2.
