# Supplementary material for: Urine-Based Molecular Diagnostic Tests for Leishmaniasis Infection in Human and Canine Populations: A Meta-Analysis
Source: Pathogens. 2021 Feb 27;10(3):269. doi: 10.3390/pathogens10030269 (PMC7996766; doi:10.3390/pathogens10030269)

Supplementary Table S1: The QUADAS tool Item

Item	Yes	No	Unclear
1. Was the spectrum of patients representative of the patients who will receive the test in practice?	(13)	(-)	(-)
2. Were selection criteria clearly described?	(13)	(-)	(-)
3.I s the reference standard likely to correctly classify the target condition?	(13)	(-)	(-)
4. Is the time period between reference standard and index test short enough to be reasonably sure that the target condition did not change between the two tests?	(8)	(-)	(5)
5. Did the whole sample or a random selection of the sample, receive verification using a reference standard of diagnosis?	(13)	(-)	(-)
6. Did patients receive the same reference standard regardless of the index test result?	(13)	(-)	(-)
7. Was the reference standard independent of the index test (i.e., the index test did not form part of the reference standard)?	(13)	(-)	(-)
8. Was the execution of the index test described in sufficient detail to permit replication of the test?	(10)	(-)	(3)
9. Was the execution of the reference standard described in sufficient detail to permit its replication?	(13)	(-)	(-)
10. Were the index test results interpreted without knowledge of the results of the reference standard?	(-)	(3)	(10)
11. Were the reference standard results interpreted without knowledge of the results of the index test?	(13)	(-)	(-)
12. Were the same clinical data available when test results were interpreted as would be available when the test is used in practice?	(13)	(-)	(-)
13. Were uninterpretable/ intermediate test results reported?	(13)	(-)	(-)
14. Were withdrawals from the study explained?	(13)	(-)	(-)

Supplementary Table S2: The Quadas Tool 2

	Bias				Applicability		
	**PATIENT SELECTION**	**INDEX TEST**	**REFERENCE STANDARD**	**FLOW AND TIMING**	**PATIENT SELECTION**	**INDEX TEST**	**REFERENCE STANDARD**
Mirzaei	Low	Low	Low	Low	Low	Low	Low
Mirzaei	Low	Low	Low	Low	Low	Low	Low
Da Costa Lima Junior	Low	High	Low	Low	Low	Low	Low
Pessoa-E-Silva	Low	Low	Low	Low	Low	Low	Low
Almerice Lopes da Silva	Low	Low	Low	Low	Low	Low	Low
Hernández	Low	Low	Low	Low	Low	Low	Low
Phumee	Low	Low	Low	Low	Low	Low	Low
Veland	Low	High	Low	Low	Low	Low	Low
Fisa	Low	Low	Low	Low	Low	Low	Low
Motazedian	Low	Low	Low	Low	Low	Low	Low
Manna	Low	Low	Low	Low	Low	Low	Low
Solano-Gallego	Low	Unclear	Low	Low	Low	Low	Low
Francesch	Low	High	Low	Low	Low	Low	Low

Supplementary Figure S1: The Quadas Tool 2 graph

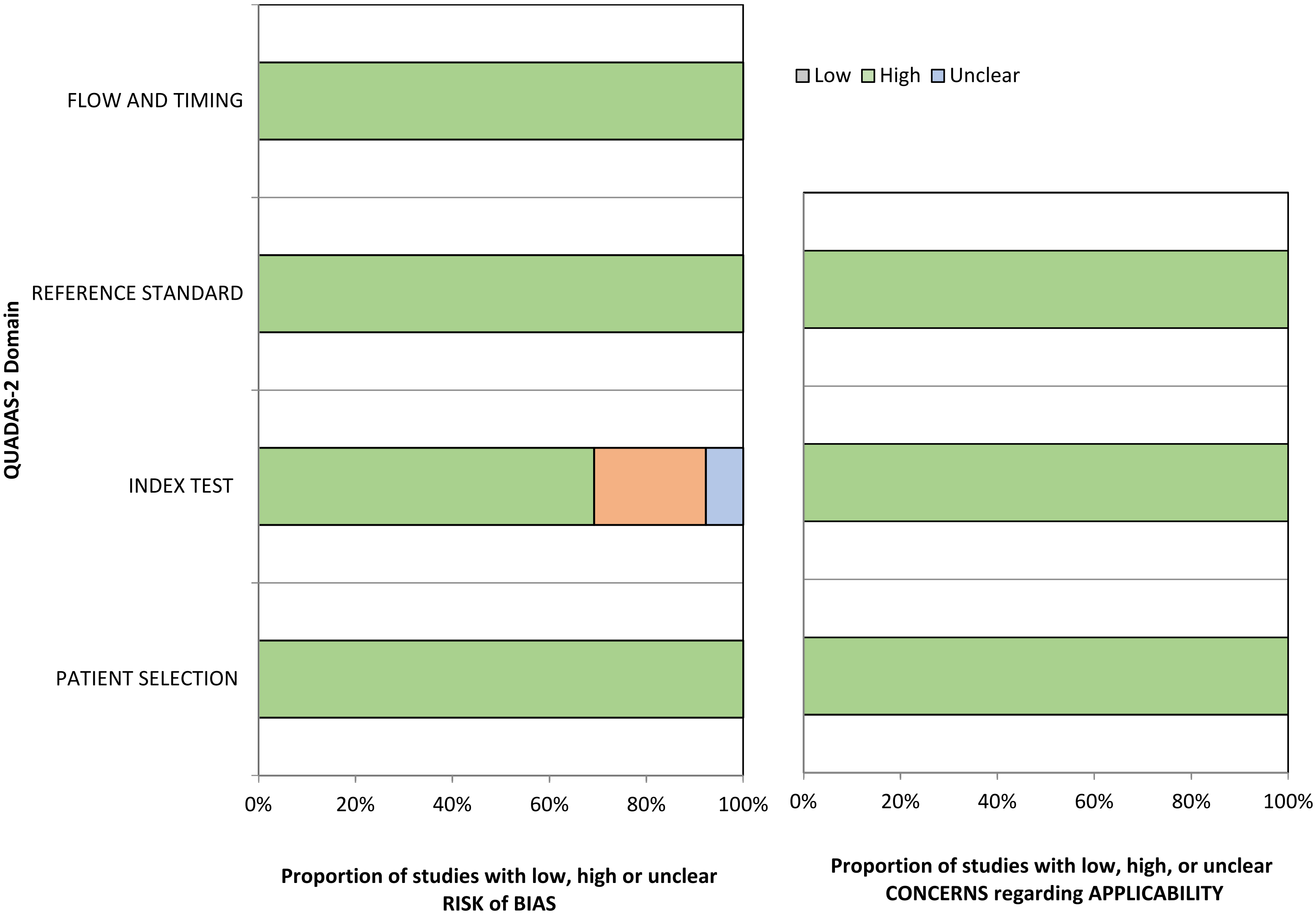

Supplement: Supplementary file 1 [file pathogens-10-00269-s001.pdf]
